# Dietary profile of pediatric obstructive sleep apnea patients, effects of routine educational counseling, and predictors for outcomes

**DOI:** 10.3389/fpubh.2023.1160647

**Published:** 2023-06-12

**Authors:** Hai-Hua Chuang, Rong-Ho Lin, Jen-Fu Hsu, Li-Pang Chuang, Hsueh-Yu Li, Tuan-Jen Fang, Yu-Shu Huang, Albert C. Yang, Guo-She Lee, Terry B. J. Kuo, Cheryl C. H. Yang, Li-Ang Lee

**Affiliations:** ^1^Department of Family Medicine, Taipei and Linkou Main Branches, Chang Gung Memorial Hospital, Taoyuan, Taiwan; ^2^Metabolism and Obesity Institute, Taipei and Linkou Main Branches, Chang Gung Memorial Hospital, Taoyuan, Taiwan; ^3^Department of Industrial Engineering and Manage-ment, National Taipei University of Technology, Taipei, Taiwan; ^4^College of Medicine, Chang Gung University, Taoyuan, Taiwan; ^5^School of Medicine, National Tsing Hua University, Hsinchu, Taiwan; ^6^Department of Pediatrics, Linkou Main Branch, Chang Gung Memorial Hospital, Taoyuan, Taiwan; ^7^Department of Pulmonary and Critical Care Medicine, Linkou Main Branch, Chang Gung Memorial Hospital, Taoyuan, Taiwan; ^8^Sleep Center, Linkou Main Branch, Chang Gung Memorial Hospital, Taoyuan, Taiwan; ^9^Department of Otorhinolaryngology – Head and Neck Surgery, Linkou Main Branch, Chang Gung Memorial Hospital, Taoyuan, Taiwan; ^10^Department of Child Psychiatry, Linkou Main Branch, Chang Gung Memorial Hospital, Taoyuan, Taiwan; ^11^Department of Psychiatry, Taipei Veter-ans General Hospital, Taipei, Taiwan; ^12^Institute of Brain Science, National Yang Ming Chiao Tung University, Taipei, Taiwan; ^13^Faculty of Medicine, National Yang-Ming University, Taipei, Taiwan; ^14^Department of Otolaryngology, Ren-Ai Branch, Taipei City Hospital, Taipei, Taiwan; ^15^Tsaotun Psychiatric Center, Ministry of Health and Wel-fare, Nantou, Taiwan; ^16^Sleep Research Center, National Yang Ming Chiao Tung University, Taipei, Taiwan

**Keywords:** adenotonsillectomy, children, dietary profile, dietary educational counseling, food frequency, food literacy, obstructive sleep apnea, outcome assessment

## Abstract

**Background:**

Dietary behavior is a main contributing yet modifiable factor to the body weight status of children and may be involved in the pathophysiology of childhood obstructive sleep apnea (OSA). This study aimed to investigate the dietary profile of pediatric OSA patients, effects of educational counseling after adenotonsillectomy, and predictor for disease resolution.

**Methods:**

This observational study included 50 pediatric OSA patients undergoing adenotonsillectomy with routine educational counseling (Group 1), 50 pediatric OSA patients undergoing adenotonsillectomy without formal educational counseling (Group 2), and 303 healthy children without OSA (Control). The three groups were matched by age. The consumption frequency of 25 food items/groups was assessed by the Short Food Frequency Questionnaire. Quality of life was evaluated by the OSA-18 questionnaire. Sleep architecture and OSA severity were measured by standard polysomnography. Between- and within-group comparisons were analyzed by non-parametric approaches and generalized estimating equations. Prediction of disease recovery was performed by multivariable logistic regression models.

**Results:**

Group 1 children consumed fruit drinks with sugar, vegetables, sweets, chocolate, rice, and noodles more frequently than Control Group children. At baseline, the distributions of sex, weight status, OSA-18 scores, and polysomnographic variables were comparable between Group 1 and Group 2. After a 12-month follow-up, Group 1 had better improvements in physical suffering, caregiver concerns, sleep architecture, and mean peripheral oxygen saturation compared to Group 2. Furthermore, Group 1 no longer had excessive consumption of fruit drinks with sugar, chocolate, and noodles; however, food consumption frequencies did not change significantly. Notably, younger age and reduced intake of butter/margarine on bread and noodles were independent predictors of cured OSA in Group 1.

**Conclusion:**

The present study preliminarily characterized an unhealthy dietary profile among pediatric OSA patients and suggested that routine educational counseling in addition to adenotonsillectomy yielded some clinical benefits. Certain items/groups of food frequencies may be associated with disease recovery and further investigations are warranted.

## 1. Introduction

Obstructive sleep apnea (OSA) is one of the most common sleep disorders in children, with a prevalence of approximately 4% worldwide (1). In addition to snoring and abnormal breathing during sleep, childhood OSA can be comorbid with many diseases, such as obesity (2), metabolic syndrome (3), cardiovascular disease (4), and attention-deficit/hyperactivity disorder (5). Hypertrophy of the adenoid and tonsils and overweight/obesity are frequently encountered in pediatric OSA patients (6–8). Adenotonsillectomy remains the first-line treatment for childhood OSA (6, 9). However, residual OSA after adenotonsillectomy (defined by a postoperative apnea-hypopnea index [AHI] ≥ 1.0 events/h) is not uncommon, accounting for 49% of the overall adenotonsillectomy-treated pediatric OSA patients and 66% of those with obesity (10). Furthermore, age and obesity are two well-recognized risk factors for residual disease after adenotonsillectomy among pediatric OSA patients (8, 11, 12). Although residual OSA does not necessarily represent a treatment failure, efforts are underway to identify successful strategies for curing OSA.

Evidence has indicated negative impacts of inadequate or poor-qualitied sleep on weight status and metabolic prolife (13–15). Preadolescents with longer sleep duration had lower body fat percentage and better insulin sensitivity (16). Sleep restriction increases levels of ghrelin, the “hunger hormone” which promotes appetite and stimulates eating (17). Food intake following sleep restriction contains an average of 328 more calories, primarily from carbohydrates (17). Sleep duration reduction, poor sleep quality, and circadian rhythm dysregulation can all lead to insulin resistance, a cardinal pathway to the development of metabolic syndrome and type 2 diabetes (18). Some study also suggests that lack of sleep has a direct negative effect on physical activity (19). Despite still some knowledge gap in pathophysiology, it has been quite evident that insufficient or bad sleep leads to weight gain and metabolic deterioration.

Intriguingly, albeit a significant improvement in sleep quality, pediatric OSA patients are likely to increase their body mass index (BMI) z-score (20) after adenotonsillectomy. Furthermore, the velocity and amount of weight gain in turn becomes independent risk factors for OSA recurrence in children (21). Some specific foods have been associated with excessive weight gain among children and adolescents, such as butter/margarine spread, potato chips, coated fish, processed meats, desserts and sweets, milk, and savory snacks (22). Increases in caloric and protein intake also have been linked to somatic growth in this population (20). Multidisciplinary weight loss interventions have been shown to improve the severity of OSA in children and adolescents with obesity (23). However, the relationships between OSA and eating behavior remain unclear in adults (24), and data for pediatric OSA patients are even scantier (25). Evidence on the effectiveness of educational counseling is lacking. The associations of baseline characters or changes in dietary behavior with OSA treatment outcomes are unknown.

We hypothesized that pediatric OSA patients were more prone to unhealthy eating, and educational counseling would have positive effects on their dietary behavior and clinical health outcomes; furthermore, changes in eating were causally attributable to changes in subjective and objective sleep parameters after adenotonsillectomy. This study aimed to: (1) delineate the dietary profile of pediatric OSA patients and compare it to children without OSA, (2) investigate the effects of educational counseling on food consumption and sleep outcomes among pediatric OSA patients undergoing adenotonsillectomy, and (3) identify predictors for cured OSA in patients receiving combined treatment.

## 2. Materials and methods

### 2.1. Study participants

The retrospective case–controlled study retrieved data from our previously established databank (26), a collection of electronic medical records of 396 children who were referred to the Department of Otorhinolaryngology-Head and Neck Surgery at Chang Gung Memorial Hospital (Linkou Main Branch, Taoyuan, Taiwan) to treat OSA between January 2010 and August 2019. This retrospective study was approved by the Institutional Review Board of Chang Gung Medical Foundation (No. 202000873B0). The requirement for written informed consent had been waived because the subsequent analyses were based on existing data. All procedures complied with the Declaration of Helsinki 1975, and the Strengthening the Reporting of Observational Studies in Epidemiology—Nutritional Epidemiology guidelines were followed (27). Parts of the subjects’ characteristics have also been reported elsewhere (7, 8, 28–31).

The inclusion criteria of this study were: (1) age 5–12 years, (2) available polysomnography confirming the diagnosis of OSA based on the definition of obstructive AHI ≥ 2.0 events/h or obstructive apnea index ≥1.0 event/h (8, 32), and (3) a history of adenotonsillectomy at our department during the study period. The exclusion criteria were: (1) patients with craniofacial, neuromuscular, or chronic inflammatory disorders that required multi-modality treatments (28, 33), and (2) those with no available data of item/group-specific food frequencies, quality of life, or polysomnographic data at baseline and 6 months post-adenotonsillectomy.

The subjects were further divided into ‘Group 1’ (adenotonsillectomy with routine educational counseling) and ‘Group 2’ (adenotonsillectomy without formal educational counseling). Furthermore, Group 1 and Group 2 participants were matched by age, sex, BMI z-score, AHI, and follow-up period.

To compare the dietary profiles of the study participants to the general population, we included data from a prospective internet survey investigating item/group-specific food frequencies among healthy Taiwanese children without obvious OSA symptoms. We invited parents and caregivers who had children without habitual snoring to evaluate their children’s food frequencies from November 24, 2022, to November 30, 2022. The inclusion criteria were: (1) age 5–12 years, (2) no obvious OSA symptoms such as habitual snoring, sleep-disturbed breathing, chronic mouth breathing, daytime sleepiness, and attention-deficit/hyperactivity, and (3) total score of the OSA-18 questionnaire <60 (34). The exclusion criteria were: (1) any known history of chronic disorders such as neuromuscular, gastrointestinal, or cardiovascular disorders, and (2) any long-termed usage of medications. All participants filled in the questionnaire voluntarily without any incentive offered. The Institutional Review Board of Chang Gung Medical Foundation approved this internet survey (No. 202201649B0), and the anonymous volunteers (children’s parents/caregivers) agreed to participate after reading the informed consent.

### 2.2. Measurements

#### 2.2.1. Clinical information

Age, sex, tonsil size, adenoidal-nasopharyngeal ratio (ANR), and BMI z-score were recorded. Obesity was defined by a BMI z-score ≥ 1.645 (35). Radical adenotonsillectomy procedures, including extracapsular tonsillectomy and adenoidectomy, were performed by the principal investigators in a single stage under general anesthesia (36). All the children received inpatient care with an average hospitalization of 3 days and ate a regular diet within 2 weeks.

#### 2.2.2. Food frequency

All caregivers recorded the consumption frequency of 23 food items in their children using the Short Food Frequency Questionnaire (SFFQ) (37). Nine questions of the SFFQ pertain to drinks, including full-fat milk, low-fat milk (1.5% fat), semi-skimmed milk (0.7% fat), skimmed milk, orange juice, fruit drinks and soft drinks with or without sugar. The SFFQ also contains questions on each of the following food items or food groups: potatoes, vegetables, fruit/berries, potato chips, whole meal bread, fish for dinner (not including bread spread), pizza, hamburgers/hot dogs/kebabs, sweets, chocolate, savory snacks, peanuts and cod liver oil/vitamin supplements, and an additional question on the use of butter/margarine on bread. In addition, two Chinese food items (rice and noodles) were also investigated in the present study. The participants and their caregivers were asked to recall their food habits in the past 1 month and filled in the questionnaire together. The frequency scale used for drinks was 0 (never-seldom), 1 (1–3 glasses per month), 2 (1–3 glasses per week), 3 (4–6 glasses per week), 4 (1–3 glasses per day), 5 (4–6 glasses per day), and 6 (7 glasses or more per day). Half a liter was defined as being equal to 3 glasses. For other foods, the frequency scales were 0 (never-seldom), 1 (1–3 times per month), 2 (1–3 times per week), 3 (4–6 times per week), 4 (1 time per day), 5 (2 times per day), 6 (3 times per day), and 7 (4 or more times per day). The question about the use of butter/margarine on bread was answered with 1 (yes) or 0 (no). The reproducibility of the 23 food items/groups of the SFFQ ranged from moderate to almost perfect (intraclass correlation coefficients: 0.58–0.84) (38) with moderate test–retest reliability (39). It has been validated to rank children according to food item/group intake (38), dietary behaviors (40, 41), and adherence to dietary guidelines (42).

#### 2.2.3. Quality of life

All caregivers evaluated their children’s OSA-related quality of life using the Chinese version of the OSA-18 questionnaire (43), which includes 18 items scored using a 7-point ordinal scale (overall range, 18–126) and has been shown to have excellent test–retest reliability (34). This questionnaire collected information about five domains that are considered to be elements in the quality of life: sleep disturbance (4 items), physical suffering (4 items), emotional distress (3 items), daytime problems (3 items), and caregiver concerns (4 items).

#### 2.2.4. Polysomnography

All participants underwent full-night, in-laboratory polysomnography (Nicolet Biomedical Inc., Madison, WI, United States) to document objective sleep characteristics (33). Total sleep time, sleep stages, AHI, apnea index, arousal index, mean peripheral oxygen saturation (SpO_2_), and minimal SpO_2_ were scored and manually verified by the study investigators, according to the 2012 American Academy of Sleep Medicine Scoring Manual (44). An apnea episode was defined as a ≥ 90% decrease in airflow for a duration of ≥2 consecutive breaths, and a hypopnea episode was defined as a ≥ 30% decrease in airflow in association with electroencephalographic arousal or a ≥ 3% reduction in SpO_2_ for a duration of ≥2 consecutive breaths. The AHI was calculated by dividing the sum of all apneas and hypopneas by the hours of total sleep time. Herein, ‘cured OSA’ was defined as a reduction in both obstructive AHI < 2.0 events/h and obstructive apnea index <1.0 event/h after adenotonsillectomy and/or educational counseling (32).

### 2.3. Routine educational counseling

Each child of Group 1, who had been enrolled in our previous study [CMRPG3F1091-3, approved by the Institutional Review Board of Chang Gung Medical Foundation (No. 201507279A3), study period 2016–2019] (8, 28), received three sessions of age-appropriate educational counseling within 3 months after adenotonsillectomy. The children and their caregivers were given verbal recommendations for sleep hygiene (adequate sleep, early sleep, avoid caffeine after lunchtime, avoid large meals or vigorous physical activity before bedtime), healthy eating [limit sweetened beverages, eat less fast food, recommend the Daily Dietary Guidelines of Taiwan for children as a reference material (45)], regular exercise (increase outdoor/after-school exercise, exercise training), and nasal saline irrigation (46). Each session of educational counseling was conducted face-to-face for 10–15 min by the research investigators and assistants.

### 2.4. Statistical analysis

Data were analyzed using G*Power 3.1.9.2 (Heinrich-Heine University, Dusseldorf, Germany), SPSS version 25.0 (IBM Corp., Armonk, NY, United States), and GraphPad Prism 9.0 for Windows (Graph Pad Software Inc., San Diego, CA, United States). The Shapiro–Wilk test was used to examine normality, which showed that most of the continuous variables of interest were non-parametric. Therefore, descriptive statistics were expressed as median (interquartile range [IQR]) for continuous and skewed variables and number (proportion) for categorical variables.

Based on a previous study (47), we applied fruit frequency to estimate the sample size. The modal scores of fruit consumption in healthy children without OSA (*n* = 177), pediatric OSA patients without obesity (*n* = 25), and pediatric OSA patients with obesity (*n* = 43) were 3–5 times/week, 3–5 times/week, and 1–2 times/week, respectively (Kruskal-Wallis test of variance by ranks, H(2245) = 7.0, *p* = 0.03). To compare the difference between healthy children without OSA and pediatric OSA patients, we estimated the weighted modal score of pediatric OSA patients. To reach 95% power with a type I error of 0.05, the total sample size would be 38. However, the investigation was powered to detect item/group-specific food frequencies as low as 0% reliably; we estimated the absence of item/group-specific food frequencies among 300 children would generate a one-sided 97.5% confidence interval between 0 and 0.99% (48).

For continuous and skewed variables, the Mann–Whitney *U* test was used to assess between-group differences and changes, and the Wilcoxon signed-rank test was used to assess within-group changes, as appropriate. Differences in independent categorical variables between two subgroups were analyzed using Fisher’s exact test, and differences in related categorical variables within groups were assessed using the McNemar test. Relationships between variables of interest were assessed using Spearman and point-biserial correlation tests, as appropriate.

Multivariable logistic regression models, including variables with a *p*-value < 0.200 (49), with manual selection based on a probability of *F* < 0.05 were used to identify independent variables for predicting cured OSA using a parsimonious approach. The variance inflation factor (VIF) of each predictor was calculated to adjust for intervariable relationships within the model. The regression model was repeated after removing all variables with a VIF ≥ 5 to reduce multicollinearity (50).

To compare differences in the changes of outcome variables by post-intervention OSA status, generalized estimating equations with adjustments for pre-intervention age, sex, and obesity were used. Specifically, each subgroup variable was entered into separate generalized estimating equations that included the main effects of time (post-intervention vs. pre-intervention) and subgroup variable (e.g., boy vs. girl), a two-way interaction term of time × subgroup variable, and the main effects of baseline characteristics. A two-sided *p*-value of <0.05 was considered statistically significant.

## 3. Results

### 3.1. Groups of the study and between-group comparisons

**Figure 1:**
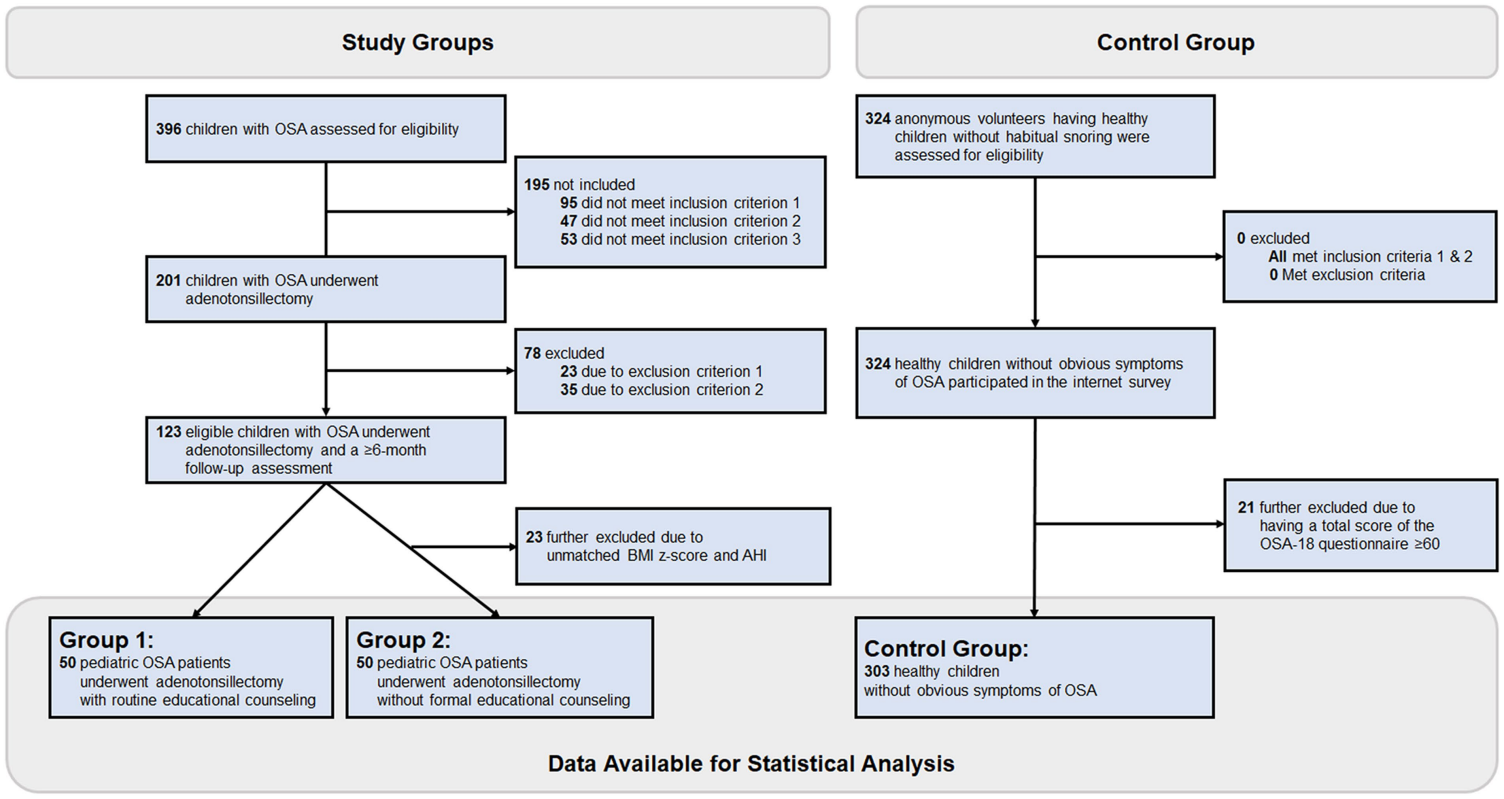
Flowchart of this observation study. Two-hundred one of 396 eligible children with OSA met the inclusion criteria of the study groups. Among them, 78 were excluded due to the exclusion criteria, and 23 were further excluded due to being unmatched by the AHI and BMI z-score between Group 1 (adenotonsillectomy with routine educational counseling) and Group 2 (adenotonsillectomy without formal educational counseling). Besides, 324 anonymous volunteers having healthy children without habitual snoring met the inclusion and exclusion criteria of the control group, and 21 were further excluded due to having a total score of the OSA-18 questionnaire ≥60. After that, the Control Group included 303 children without obvious OSA symptoms. AHI, apnea-hypopnea index; BMI, body mass index; OSA, obstructive sleep apnea.

Figure 1 demonstrates the flowchart of the present study. Fifty pediatric OSA patients (38 [76%] boys and 12 [24%] girls) with a median (IQR) age of 7.0 (5.8–10.0) years undergoing adenotonsillectomy with routine educational counseling (Group 1), 50 pediatric OSA patients (40 [80%] boys and 10 [20%] girls) with a median age of 7.4 (5.9–10.3) years undergoing adenotonsillectomy without formal educational counseling (Group 2), and 303 healthy children without obvious OSA symptoms (Control Group) were included for further analysis.

The Control Group in total had 303 healthy children (159 [52%] boys and 144 [48%] girls; median total OSA-18 score of 35 [28–43]), consisting of 45 (14.9%) 5-year-old, 44 (14.5%) 6-year-old, 39 (12.9%) 7-year-old, 43 (14.2%) 8-year-old, 38 (12.5%) 9-year-old, 45 (14.9%) 10-year-old, and 49 (16.2%) 11-year-old children. The median scores of sleep disturbance, physical suffering, emotional distress, daytime problems, and caregiver concerns of the OSA-18 questionnaire of the Control Group were 6 (5–8), 8 (5–11), 7 (4–9), 6 (4–9), and 7 (4–9), respectively. The difference in age between Group 1 and the Control Group was not significant (*p* = 0.161); however, the proportion of boys in Group 1 was significantly higher than that of the Control Group (*p* = 0.002). Because scores of the OSA-18 questionnaire and scales of the SFFQ questionnaire between boys and girls were comparable in Group 1 and Control Group, we did not perform further weighting procedures in the following statistical analyses.

Table 1 shows item/group-specific food frequencies in Group 1 (at baseline and post-adenotonsillectomy) and Control Group (at base line). In Group 1, the most consumed drinks in descending order of frequency were full-fat milk, orange juice and fruit drinks with sugar. The main consumed foods in descending order of frequency included vegetables, rice, fruit/berries, noodles, fish for dinner, and sweets. In the Control Group, all item/group-specific food frequencies between boys and girls were comparable. The most consumed drinks were full-fat milk, fruit drinks with sugar and soft drinks with sugar. The main items/groups of other foods included vegetables, rice, fruit/berries, fish for dinner, savory snacks, and noodles. In addition, Group 1 children ate fruit drinks with sugar, vegetables, sweets, chocolate, rice, and noodles more frequently than the Control Group children did. However, Group 1 children’s daily consumption of vegetables and fruit/berries were significantly lower than those of Taiwanese children aged 7–12 years (vegetables: 1.8 times/day; fruits: 1.0 time/day) (*p* = 0.013 and < 0.001, respectively) (51).

**Table 1:** Item/group-specific food frequencies in Group 1 (at baseline and post-intervention) and Control Group (baseline).

	Group 1				Control Group		
	Before	After					
Items/Groups	*N* = 50	*N* = 50	Changes	*p*^a^	*N* = 303	*p*^b^	*p*^c^
Full-fat milk (scale)	2 (1–3)	2 (2–3)	0 (0–1)	0.075	2 (1–3)	0.677	0.347
Low-fat milk (scale)	0 (0–0)	**0 (0–0)**	0 (0–0)	0.168	**0 (0–0)**	0.316	**0.009**
Semi-skimmed milk (scale)	0 (0–0)	0 (0–0)	0 (0–0)	0.317	0 (0–0)	0.995	0.316
Skimmed milk (scale)	0 (0–0)	0 (0–0)	0 (0–0)	0.414	0 (0–0)	0.548	0.889
Orange juice (scale)	1 (0–1)	0 (0–1)	0 (−1–0)	0.564	0 (0–1)	0.082	0.393
Fruit drink with sugar (scale)	**1 (1–2)**	1 (0–1)	0 (−1–0)	0.130	**1 (0–1)**	**0.020**	0.544
Fruit drink without sugar (scale)	0 (0–1)	0 (0–1)	0 (0–0)	0.833	0 (0–1)	0.860	0.640
Soft drinks with sugar (scale)	0 (0–1)	1 (0–2)	0 (0–1)	0.679	1 (0–1)	0.928	0.398
Soft drinks without sugar (scale)	0 (0–0)	0 (0–0)	0 (0–0)	0.257	0 (0–1)	0.193	0.979
Boiled potatoes (scale)	0 (0–0)	0 (0–1)	0 (0–0)	0.819	0 (0–1)	0.634	0.847
Potato chips (scale)	1 (0–1)	1 (1–1)	0 (0–0)	0.371	1 (0–1)	0.490	0.139
Vegetables (scale)	**5 (4–5)**	**5 (3–5)**	0 (−1–0)	0.276	**4 (3–5)**	**<0.001**	**0.009**
Fruit/berries (scale)	3 (2–4)	3 (2–4)	0 (−1–1)	0.951	3 (2–4)	0.692	0.761
Whole meal bread (scale)	1 (0–1)	1 (0–1)	0 (0–0)	0.701	0 (0–1)	0.368	0.446
Fish for dinner (scale)	2 (1–3)	2 (1–3)	0 (−1–1)	0.544	2 (1–3)	0.567	0.798
Pizza (scale)	0 (0–1)	0 (0–1)	0 (0–1)	0.072	1 (0–1)	0.170	0.572
Hamburgers/hot dogs/kebabs (scale)	1 (0–1)	1 (0–1)	0 (0–1)	0.285	1 (0–1)	0.633	0.105
Sweets (scale)	**2 (1–2)**	**1 (1–2)**	0 (−1–0)	0.592	**1 (1–2)**	**<0.001**	**0.009**
Chocolate (scale)	**1 (1–2)**	1 (0–2)	0 (−1–0)	0.333	**1 (0–1)**	**<0.001**	0.085
Savory snacks (scale)	1 (1–2)	1 (1–2)	0 (−1–1)	0.880	2 (1–2)	0.128	0.098
Peanuts (scale)	0 (0–1)	0 (0–1)	0 (0–0)	0.549	0 (0–1)	0.124	0.180
Cod liver oil/vitamin supplements (scale)	0 (0–1)	0 (0–1)	0 (0–0)	0.166	0 (0–2)	0.257	0.435
Butter/margarine on bread (yes)	21 (42)	23 (46)	0 (0–0)	0.815	124 (42)	>0.999	0.540
Rice (scale)	**5 (5–5)**	**5 (4–5)**	0 (−1–0)	0.371	**3 (3–5)**	**<0.001**	**<0.001**
Noodles (scale)	**3 (2–4)**	3 (2–3)	0 (−1–1)	0.554	**2 (2–3)**	**0.048**	0.136

Results are presented as median (interquartile range) for continuous variables and as absolute number (relative frequency) for categorical variables. Bold font indicates statistically significant differences (*p* < 0.05). ^a^ Within-group comparisons were performed using the related-samples Wilcoxon signed-rank test for continuous variables or related-samples McNemar test for categorical variables, as appropriate. ^b^ Data between values before the intervention and normal values were compared using the Mann–Whitney *U* test for continuous variables or Fisher’s exact test for categorical variables, as appropriate. ^c^ Data between values after the intervention and normal values were compared using the Mann–Whitney *U* test for continuous variables or Fisher’s exact test for categorical variables, as appropriate.

Table 2 shows patient characteristics, scores of the OSA-18 questionnaire, and polysomnographic variables of Group 1 and Group 2 at baseline and post-adenotonsillectomy ≥6 months. At baseline, all variables were comparable between the two groups. The median scores of sleep disturbance, physical suffering, emotional distress, daytime problems, caregiver concerns, and total scores of the OSA-18 questionnaire of Group 1 and Group 2 (Table 2) were significantly higher than those of the Control Group.

**Table 2:** Demographic metrics and sleep variables of Group 1 and Group 2 at baseline and post-intervention ≥6 months.

	Group 1	Group 2	
	Adenotonsillectomy with routine educational counseling	Adenotonsillectomy without formal educational counseling	
Variables	*N* = 50	*N* = 50	*p*^a^
Patients’ characteristics			
Age (year)	7.0 (5.8–10.0)	7.40 (5.9–10.3)	0.493
Sex (girls/boys)	12/38	10/40	0.810
BMI z-score			
Baseline	**0.685 (−0.475–2.100)**	**1.370 (0.205–2.110)**	0.203
Post-AT	**1.305 (−0.218–2.173)**	**2.000 (1.260–2.289)**	**0.010**
Change	0.135 (−0.023–0.713)	0.190 (0.010–0.873)	0.503
*p*-value^b^	**0.002**	**0.001**	
Tonsil size (grade)	3 (3–4)	3 (3–4)	0.359
ANR	0.791 (0.665–0.869)	0.826 (0.760–0.850)	0.149
OSA-18 questionnaire			
Sleep disturbance (score)			
Baseline	**18 (15–22)**	**19 (17–23)**	0.391
Post-AT	**9 (8–11)**	**9 (7–11)**	0.639
Change	−10 (−13–−5)	−10 (−14–−7)	0.600
*p*-value^b^	**<0.001**	**<0.001**	
Physical symptoms (score)			
Baseline	**17 (15–19)**	**18 (15–21)**	0.699
Post-AT	**11 (8–13)**	**11 (9–16)**	0.092
Change	−7 (−11–−3)	−7 (−9–−2)	0.356
*p*-value^b^	**<0.001**	**0.001**	
Emotional distress (score)			
Baseline	**10 (7–13)**	**11 (8–12)**	0.918
Post-AT	**8 (5–10)**	**6 (5–8)**	0.434
Change	−3 (−5–−1)	−3 (−6–−2)	0.596
*p*-value^b^	**<0.001**	**0.001**	
Daytime problems (score)			
Baseline	**11 (9–14)**	**12 (10–13)**	0.974
Post-AT	**8 (6–10)**	**8 (6–10)**	0.891
Change	−3 (−6–0)	−4 (−6–−2)	0.575
*p*-value^b^	**<0.001**	**0.001**	
Caregiver concerns (score)			
Baseline	**18 (15–22)**	**18 (15–22)**	0.741
Post-AT	**8 (6–11)**	**9 (7–12)**	**0.036**
Change	−10 (−14–−6)	−8 (−11–−4)	0.137
*p*-value^b^	**<0.001**	**0.001**	
Total score			
Baseline	**77 (69–91)**	**77 (64–86)**	0.362
Post-AT	**53 (43–60)**	**44 (36–54)**	0.199
Change	−31 (−47–−19)	−33 (−41–−18)	0.986
*p*-value^b^	**<0.001**	**<0.001**	
Polysomnography			
Total sleep time (minutes)			
Baseline	336 (304–351)	324 (296–350)	0.164
Post-AT	340 (320–351)	344 (322–349)	0.680
Change	2 (−17–36)	12 (−7–43)	0.311
*p*-value^b^	0.377	0.056	
N1 sleep (%)			
Baseline	10.3 (6.2–17.3)	**9.6 (7.4–14.7)**	0.986
Post-AT	9.7 (7.0–14.4)	**8.6 (4.6–12.0)**	0.061
Change	−0.1 (−6.4–6.2)	−2.5 (−8.8–2.3)	0.243
*p*-value^b^	0.383	**0.025**	
N2 sleep (%)			
Baseline	**40.2 (31.4–45.2)**	38.3 (32.6–42.9)	0.398
Post-AT	**43.0 (37.4–48.2)**	41.1 (36.3–47.2)	0.291
Change	2.6 (−1.6–8.6)	2.8 (−3.7–9.2)	0.850
*p*-value^b^	**0.017**	0.087	
N3 sleep (%)			
Baseline	**26.1 (22.5–32.2)**	30.3 (21.9–35.4)	0.276
Post-AT	**24.0 (20.1–27.8)**	**29.6 (23.3–35.2)**	**0.002**
Change	−1.9 (−8.9–1.4)	−1.3 (−10.5–10.3)	0.071
*p*-value^b^	**0.006**	0.577	
REM sleep (%)			
Baseline	**19.2 (13.3–22.7)**	20.2 (16.0–22.6)	0.243
Post-AT	**21.3 (17.4–25.3)**	19.7 (16.1–21.8)	0.163
Change	3.7 (−3.6–9.6)	2.1 (−3.7–5.0)	0.117
*p*-value^b^	**0.006**	0.497	
AHI (events/h)			
Baseline	**8.8 (4.1–23.3)**	**15.4 (7.5–29.7)**	0.128
Post-AT	**2.9 (1.9–5.5)**	**3.8 (1.7–9.0)**	0.351
Change	−6.1 (−15.5–−0.7)	−5.7 (−19.8–−0.8)	0.973
*p*-value^b^	**<0.001**	**<0.001**	
Apnea index (events/h)			
Baseline	**1.8 (1.0–4.4)**	**2.0 (0.6–7.1)**	0.764
Post-AT	**1.1 (0.2–1.6)**	**1.0 (0.4–2.3)**	0.423
Change	−0.7 (−3.4–0.0)	−0.8 (−2.9–0.5)	0.597
*p*-value^b^	**<0.001**	**0.007**	
Arousal index (events/h)			
Baseline	**10.9 (7.7–18.7)**	**12.9 (7.8–21.1)**	0.506
Post-AT	**7.9 (5.6–10.5)**	**7.6 (6.3–12.2)**	0.468
Change	−3.6 (−9.8–0.3)	−3.8 (−13.2–0.6)	0.901
*p*-value^b^	**<0.001**	**0.001**	
Mean SpO_2_ (%)			
Baseline	**97 (97–98)**	97 (96–98)	0.070
Post-AT	**98 (97–98)**	**97 (97–98)**	**0.015**
Change	0 (0–1)	0 (0–1)	0.807
*p*-value^b^	**0.009**	0.206	
Minimal SpO_2_ (%)			
Baseline	**89 (84–92)**	**86 (82–90)**	0.084
Post-AT	**91 (89–94)**	**91 (86–92)**	0.059
Change	2 (0–7)	3 (−1–7)	0.645
*p*-value^b^	**<0.001**	**<0.001**	
Snoring index (events/h)			
Baseline	312.6 (103.0–469.9)	193.2 (97.7–392.2)	0.230
Post-AT	188.6 (68.4–362.1)	131.5 (1.9–307.6)	0.092
Change	−127.5 (−346.6–149.8)	−97.8 (−274.5–170.6)	0.415
*p*-value^b^	0.072	0.386	

Results are presented as median (interquartile range) for continuous variables and as absolute number for categorical variables. Bold font indicates statistically significant differences (*p* < 0.05). ^a^ Data were compared using the independent-samples Mann–Whitney *U* test for continuous variables and Fisher’s exact test for categorical variables. ^b^ Data were compared using the related-samples Wilcoxon signed-rank test for continuous variables. AHI, apnea-hypopnea index; ANR, adenoidal-nasopharyngeal ratio; AT, adenotonsillectomy; BMI, body mass index; OSA, obstructive sleep apnea; REM, rapid eye movement; SpO_2_, peripheral oxygen saturation.

The median follow-up time of Group 1 (12 [IQR, 12–15] months) and Group 2 (15 [IQR, 8–17] months) were comparable (*p* = 0.950).

After adenotonsillectomy, BMI z-scores of both Groups 1 and 2 significantly increased. The follow-up BMI z-score of Group 1 was significantly lower than that of Group 2. Both Group 1 and Group 2 had significantly reduced scores in five domains and total scores on the OSA-18 questionnaire (Table 2). The follow-up score of caregiver concerns of Group 1 was significantly lower than that of Group 2. Follow-up scores of physical suffering (*p* = 0.057), emotional distress (*p* = 0.569), and caregiver concerns (*p* = 0.219) of Group 1 were comparable to those of the Control Group; however, scores of sleep disturbance (*p* < 0.001) and daytime problems (*p* = 0.007), and total scores (*p* < 0.001) of Group 1 were still significantly higher than those of the Control Group. Follow-up scores of sleep disturbance (*p* < 0.001), physical suffering (*p* = 0.001), daytime problems (*p* = 0.016), and caregiver concerns (*p* = 0.001), and total scores (*p* = 0.001) were significantly higher in Group 2 than in the Control Group, while follow-up scores of emotional distress were comparable between Group 2 and Control Group (*p* = 0.575).

After adenotonsillectomy, Group 1 patients had significantly higher proportions of N2 sleep and rapid eye movement (REM) sleep, mean SpO_2_ and minimal SpO_2_, and significantly lower proportion of N3 sleep, AHI, apnea index, and arousal index. Group 2 had significantly higher mean SpO_2_ and minimal SpO_2_, and significantly lower proportion of N1 sleep, AHI, apnea index, and arousal index. Group 1 had a significantly lower follow-up proportion of N3 sleep and a higher mean SpO_2_ than Group 2.

### 3.2. Within-group analysis and outcome prediction models in the Group 1

**Figure 2:**
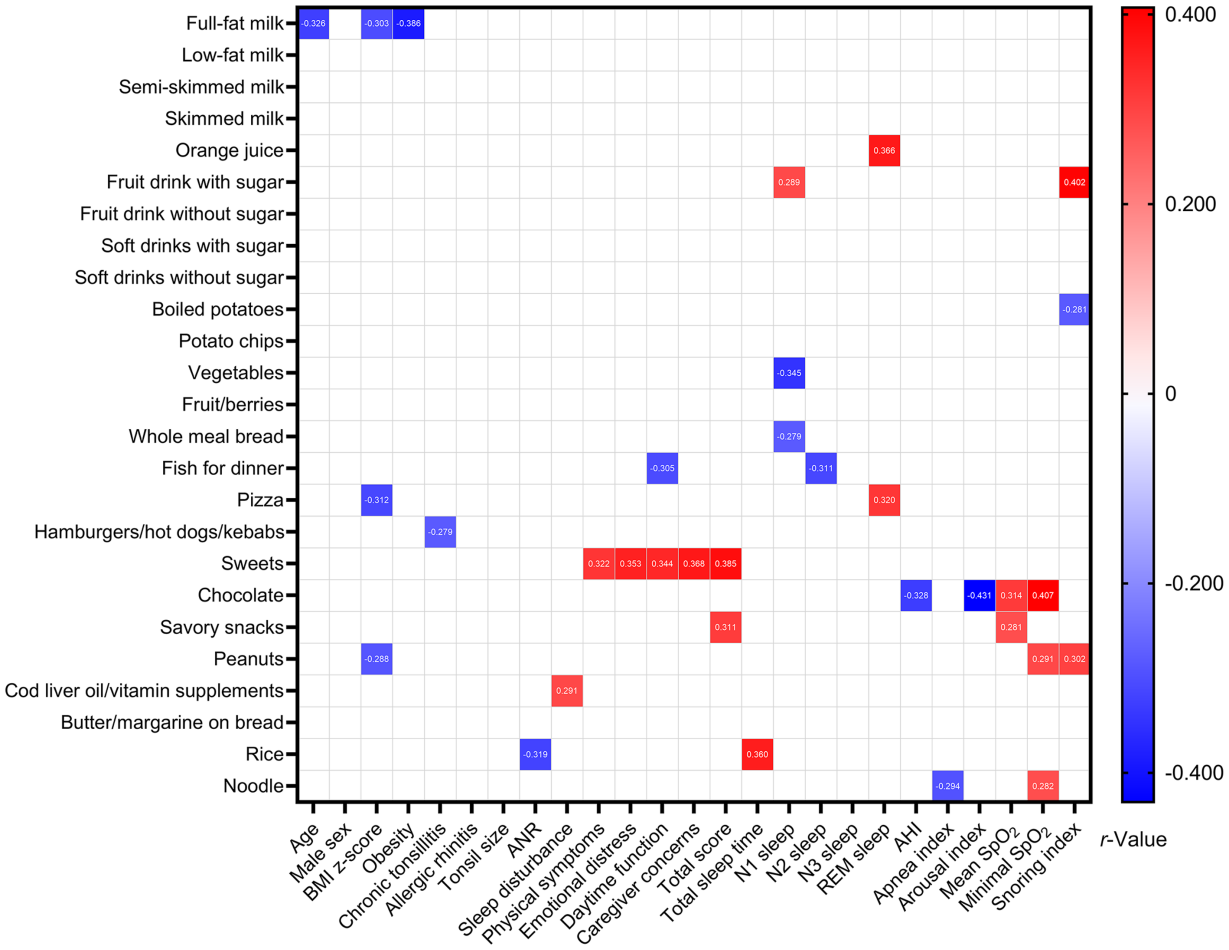
Associations between variables of interest among Group 1 at baseline. Data are summarized as Spearman’s or point-biserial *rho*, as appropriate. Blank spaces mean two-sided *p*-values ≥0.05.

Figure 2 demonstrates several significant relationships between variables of interest in Group 1 at baseline. Notably, AHI was inversely related to chocolate, whereas the apnea index was inversely associated with noodles.

Although post-adenotonsillectomy changes in item/group-specific food frequencies were not significant in Group 1, excessive consumption of fruit drinks with sugar, chocolate, and noodles no longer persisted (Table 1). However, Group 1 children drank low-fat milk and still ate vegetables, sweets, and rice more frequently than the Control Group children.

Twenty-five (50%) children had cured OSA, and 25 (50%) had residual OSA. The baseline characteristics of the two outcome subgroups are shown in Table 3. Notably, those with cured OSA were significantly younger than those who had residual OSA. The ANR of those with cured OSA was significantly higher than those who had residual OSA. Furthermore, the proportions of REM stage sleep, mean SpO_2_, and snoring index of those with cured OSA were significantly higher than those who had residual OSA. The rest of the variables of interest were comparable between the two outcome subgroups.

**Table 3:** Baseline characteristics of the children with cured OSA and those had residual OSA after intervention in Group 1.

	Cured OSA	Residual OSA	
Characteristics	*N* = 25	*N* = 25	*p*^a^
Patient characteristics			
Age (years)	**6.0 (5.0–7.0)**	**9.0 (7.0–10.5)**	**0.001**
Male sex, *n* (%)	16 (64)	22 (88)	0.095
BMI (kg/m^2^) z-score	0.380 (−0.610–1.945)	1.190 (−0.365–2.300)	0.443
Obesity, *n* (%)	11 (44)	13 (52)	0.778
Chronic tonsillitis, *n* (%)	5 (20)	5 (24)	>0.999
Allergic rhinitis, *n* (%)	19 (76)	18 (72)	>0.999
Tonsil size	3 (3–4)	3 (3–4)	0.164
ANR	**0.811 (0.736–0.882)**	**0.744 (0.634–0.844)**	**0.028**
Short food frequency questionnaire			
Full-fat milk (scale)	2 (2–3)	2 (1–3)	0.563
Low-fat milk (scale)	0 (0–0)	0 (0–0)	0.503
Semi-skimmed milk (scale)	0 (0–0)	0 (0–0)	0.317
Skimmed milk (scale)	0 (0–0)	0 (0–0)	0.317
Orange juice (scale)	1 (0–1)	1 (0–1)	>0.999
Fruit drink with sugar (scale)	1 (1–2)	1 (1–2)	0.806
Fruit drink without sugar (scale)	0 (0–1)	0 (0–1)	0.961
Soft drinks with sugar (scale)	0 (0–2)	0 (0–1)	0.501
Soft drinks without sugar (scale)	0 (0–0)	0 (0–0)	0.493
Boiled potatoes (scale)	0 (0–1)	0 (0–0)	0.472
Potato chips (scale)	1 (0–1)	1 (1–1)	0.115
Vegetables (scale)	5 (4–5)	5 (4–5)	0.251
Fruit/berries (scale)	4 (3–4)	3 (2–4)	0.165
Whole meal bread (scale)	1 (0–1)	0 (0–2)	0.859
Fish for dinner (scale)	2 (1–3)	2 (1–4)	0.108
Pizza (scale)	0 (0–1)	0 (0–1)	0.688
Hamburgers/hot dogs/kebabs (scale)	1 (0–1)	1 (0–1)	0.324
Sweets (scale)	2 (1–2)	2 (1–2)	0.875
Chocolate (scale)	1 (1–2)	1 (1–2)	0.992
Savory snacks (scale)	2 (1–2)	1 (1–3)	0.669
Peanuts (scale)	0 (0–1)	0 (0–1)	0.818
Cod liver oil/vitamin supplements (scale)	0 (0–1)	0 (0–1)	0.665
Butter/margarine on bread (yes)	0 (0–1)	0 (0–1)	0.903
Rice (scale)	5 (5–5)	5 (5–6)	0.215
Noodles (scale)	3 (2–4)	2 (2–3)	0.069
OSA–18 questionnaire			
Sleep disturbance (score)	19 (16–22)	17 (14–23)	0.366
Physical symptoms (score)	18 (15–19)	17 (13–20)	0.668
Emotional distress (score)	10 (7–12)	10 (7–16)	0.690
Daytime function (score)	11 (7–14)	12 (9–15)	0.242
Caregiver concerns (score)	19 (15–23)	17 (14–22)	0.472
Total score	86 (69–92)	76 (68–93)	0.992
Polysomnographic parameters			
Total sleep time (minutes)	337 (330–350)	333 (289–351)	0.277
N1 sleep (%)	9.4 (6.4–15.6)	10.4 (5.9–21.9)	0.607
N2 sleep (%)	40.5 (32.8–46.1)	40.1 (31.4–45.4)	0.938
N3 sleep (%)	27.5 (22.9–35.7)	26.0 (21.5–30.5)	0.720
REM sleep (%)	**20.7 (17.7–23.2)**	**16.3 (11.0–22.4)**	**0.029**
AHI (events/h)	6.1 (3.7–18.8)	14.8 (4.2–26.3)	0.308
Apnea index (events/h)	2.0 (1.3–4.5)	1.3 (0.6–4.1)	0.252
Arousal index (events/h)	9.1 (7.1–15.9)	13.9 (8.6–22.8)	0.095
Mean SpO_2_ (%)	**98 (97–98)**	**97 (97–98)**	**0.049**
Minimal SpO_2_ (%)	90 (84–92)	89 (84–92)	0.335
Snoring index (events/h)	**388.0 (175.2–485.5)**	**148.1 (25.4–430.4)**	**0.049**

Results are presented as median (interquartile range) for continuous variables and as absolute number (relative frequency) for categorical variables. Bold font indicates statistically significant differences (*p* < 0.05). ^a^ Data were compared using the Mann–Whitney *U* test for continuous variables or Fisher’s exact test for categorical variables, as appropriate. AHI, apnea-hypopnea index; ANR, adenoidal-nasopharyngeal ratio; BMI, body mass index; OSA, obstructive sleep apnea; REM, rapid eye movement; SpO_2_, peripheral oxygen saturation.

After adjustments for baseline age, sex, and obesity (Table 4), the change in fish for dinner for those with cured OSA was significantly higher than for those with residual OSA. In addition, changes in noodles and REM sleep for those with cured OSA were significantly lower than those with residual OSA. The changes in butter/margarine on bread and snoring index between the two subgroups were no longer significant after adjustment. Furthermore, changes in other variables of interest were comparable between the two subgroups before and after adjustment.

**Table 4:** Changes in the characteristics of the children with cured OSA and those had residual OSA after intervention in Group 1.

	Cured OSA	Residual OSA		
Changes in characteristics	*N* = 25	*N* = 25	*p*^a^	*p*^b^
Patient characteristic				
BMI (kg/m^2^) z-score	0.120 (−0.070–0.820)	0.170 (−0.010–0.695)	0.930	0.524
Short form food frequency questionnaire				
Full-fat milk (scale)	0 (0–1)	0 (0–1)	0.214	0.406
Low-fat milk (scale)	0 (0–0)	0 (0–0)	0.693	0.601
Semi-skimmed milk (scale)	0 (0–0)	0 (0–0)	0.317	0.306
Skimmed milk (scale)	0 (0–0)	0 (0–0)	0.556	0.177
Orange juice (scale)	0 (−1–0)	0 (−1–0)	0.395	0.320
Fruit drink with sugar (scale)	0 (−1–0)	0 (−1–0)	0.559	0.692
Fruit drink without sugar (scale)	0 (−1–0)	0 (0–1)	0.296	0.691
Soft drinks with sugar (scale)	0 (−1–1)	0 (0–1)	0.539	0.623
Soft drinks without sugar (scale)	0 (0–0)	0 (0–1)	0.332	0.530
Boiled potatoes (scale)	0 (−1–0)	0 (0–0)	0.334	0.205
Potato chips (scale)	0 (0–1)	0 (−1–0)	0.076	0.210
Vegetables (scale)	0 (−1–0)	0 (−1–1)	0.801	0.756
Fruit/berries (scale)	0 (−2–1)	0 (−1–1)	0.369	0.249
Whole meal bread (scale)	0 (0–1)	0 (−1–0)	0.151	0.210
Fish for dinner (scale)	**1 (−1–2)**	**0 (−1–1)**	0.083	**0.024**
Pizza (scale)	0 (0–1)	0 (0–1)	0.618	0.945
Hamburgers/hot dogs/kebabs (scale)	0 (0–1)	0 (−1–1)	0.188	0.865
Sweets (scale)	0 (−1–0)	0 (−1–1)	0.918	0.821
Chocolate (scale)	0 (−1–0)	0 (−1–1)	0.748	0.866
Savory snacks (scale)	0 (−1–0)	0 (−1–1)	0.237	0.184
Peanuts (scale)	0 (0–0)	0 (−1–1)	0.957	0.936
Cod liver oil/vitamin supplements (scale)	0 (0–0)	0 (0–0)	0.918	0.457
Butter/margarine on bread (yes)	**0 (−1–0)**	**0 (0–1)**	**0.046**	0.146
Rice (scale)	0 (−1–0)	0 (−1–0)	0.991	0.929
Noodles (scale)	**-1 (−2–0)**	**0 (0–1)**	**0.041**	**0.033**
OSA-18 questionnaire				
Sleep disturbance (score)	−11 (−13–−7)	−9 (−12–−3)	0.173	0.294
Physical symptoms (score)	−8 (−12–−4)	−6 (−10–−2)	0.240	0.789
Emotional distress (score)	−3 (−5–−2)	-2 (−8–1)	0.977	0.409
Daytime function (score)	−3 (−6–−5)	−3 (−6–0)	0.946	0.231
Caregiver concerns (score)	−11 (−15–−6)	−9 (−15–−5)	0.203	0.326
Total score	−33 (−47–−21)	−29 (−49–−8)	0.382	0.870
Polysomnographic parameters				
Total sleep time (minutes)	1 (−29–17)	11 (−16–58)	0.112	0.165
N1 sleep (%)	0.4 (−4.9–5.5)	−2.2 (−7.0–6.3)	0.547	0.432
N2 sleep (%)	1.5 (−5.2–7.8)	2.7 (−0.5–10.3)	0.362	0.372
N3 sleep (%)	−0.4 (−9.1–1.9)	−3.5 (−8.2–0.1)	0.229	0.362
REM sleep (%)	**2.0 (−4.2–7.1)**	**7.0 (−2.4–11.3)**	0.073	**0.022**
AHI (events/h)	−5.1 (−16.7–−1.1)	−9.9 (−16.0–0.3)	0.662	0.099
Apnea index (events/h)	−0.9 (−3.4–−0.3)	-0.3 (−3.1–0.2)	0.229	0.247
Arousal index (events/h)	−3.2 (−9.4–−1.5)	−4.3 (−10.8–0.3)	0.936	0.099
Mean SpO_2_ (%)	0 (0–1)	0 (0–1)	0.773	0.091
Minimal SpO_2_ (%)	3 (1–8)	2 (−1–5)	0.199	0.588
Snoring index (events/h)	**−187.0 (−440.0–−30.0)**	**30.4 (−227.7–294.8)**	**0.031**	0.163

Results are presented as median (interquartile range) for continuous variables and as absolute number (relative frequency) for categorical variables. Bold font indicates statistically significant differences (*p* < 0.05). ^a^ Between-group changes were compared using the Mann–Whitney *U* test for continuous variables. ^b^ Data were further compared using generalized estimating equation analyses adjusted for baseline age, sex, and/or obesity status. AHI, apnea-hypopnea index; ANR, adenoidal-nasopharyngeal ratio; BMI, body mass index; OSA, obstructive sleep apnea; REM, rapid eye movement; SpO_2_, peripheral oxygen saturation.

**Figure 3:**
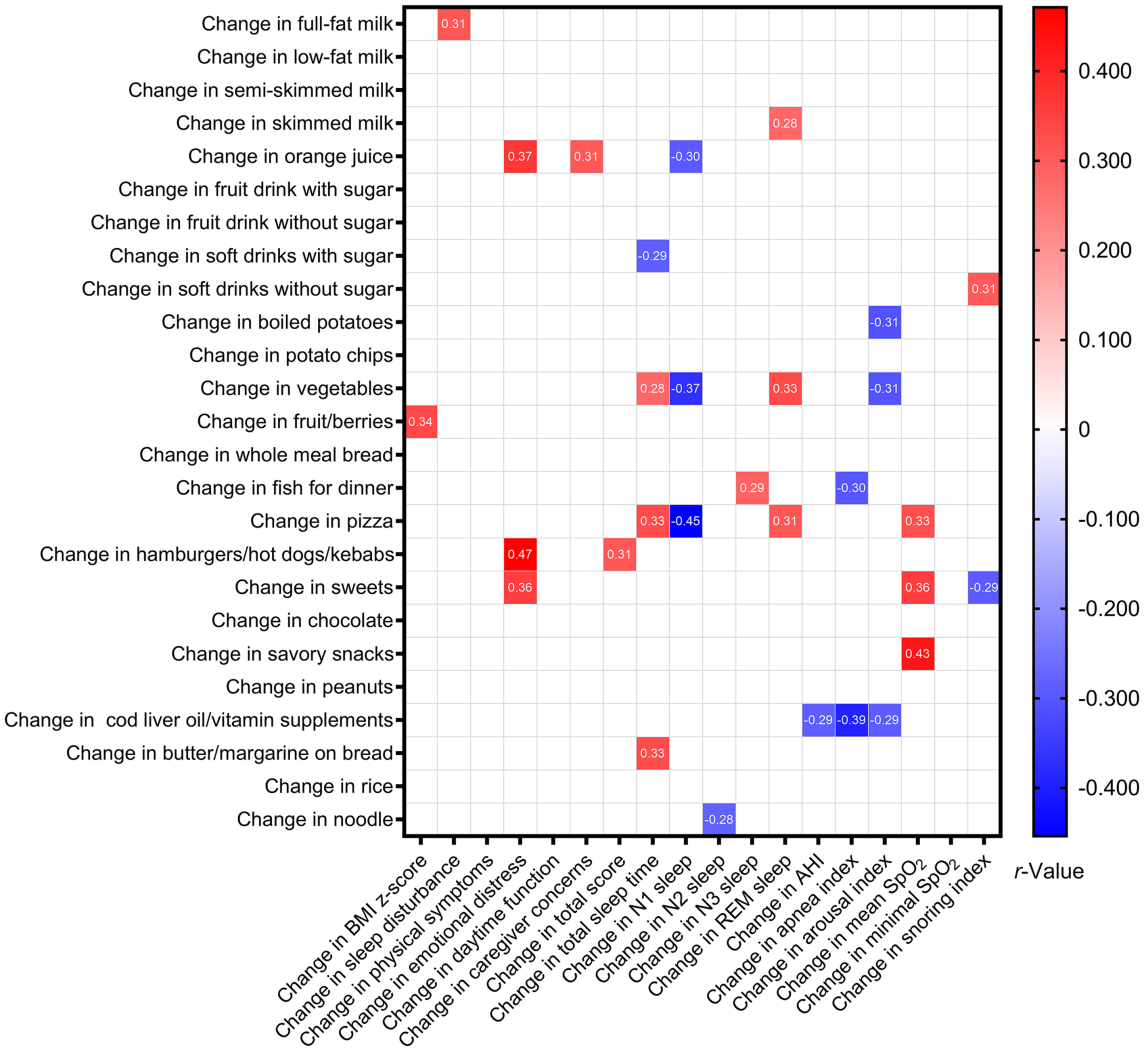
Associations between changes in variables of interest among Group 1 after intervention. Data are summarized as Spearman’s or point-biserial *rho*, as appropriate. Blank spaces mean two-sided *p*-values ≥0.05.

Significant associations were found between changes in SFFQ item/group scores and changes in BMI z-score, OSA-18 domain scores, and changes in polysomnographic parameters (Figure 3). Notably, change in AHI was inversely related to change in cod liver oil/vitamin supplements. Change in apnea index was inversely correlated with changes in fish for dinner and cod liver oil/vitamin supplements.

To construct prediction models for cured OSA, we included baseline variables with a *p*-value <0.200 (Tables 3, 4; not including changes in polysomnographic parameters), including age, male sex, tonsil size, ANR, potato chips, fruit/berries, fish for dinner, noodles, REM sleep, arousal index, mean SpO_2_, snoring index, change in skimmed milk, change in fish for dinner, change in butter/margarine on bread, and change in noodles in multivariable logistic regression analyses.

Table 5 summarizes the full model and the final parsimonious model to predict cured OSA. After removing variables with a VIF ≥ 5 (ANR, arousal index, mean SpO_2_), the full model identified age and change in butter/margarine on bread as independent predictors under the control of male sex, tonsil size, potato chips, fruit/berries, fish for dinner, noodles, REM sleep, snoring index, change in skimmed milk, change in fish for dinner, and change in noodles. The parsimonious model (including baseline clinical variables and changes in food items/groups) identified age, change in butter/margarine on bread, and change in noodles as the best predictors of cured OSA.

**Table 5:** Prediction models for cured OSA after intervention in Group 1.

Predictors	Exp(*β*) (95% CI)	*p*	VIF	*Adjusted R^2^*
Full model				
Age (years)	**0.368 (0.146–0.928)**	**0.034**	1.347	0.766
Male sex	0.079 (0.003–2.182)	0.134	1.427	
Tonsil size	7.739 (0.666–89.998)	0.102	1.370	
Potato chips (scale)	0.351 (0.025–4.997)	0.440	1.384	
Fruit/berries (scale)	1.904 (0.520–6.974)	0.331	1.424	
Fish for dinner (scale)	0.337 (0.081–1.407)	0.136	1.583	
Noodles (scale)	3.733 (0.770–18.108)	0.102	2.009	
REM sleep (%)	1.093 (0.883–1.352)	0.414	1.262	
Snoring index (events/h)	1.003 (0.997–1.009)	0.332	1.493	
Change in skimmed milk (scale)	0.880 (0.115–6.750)	0.902	1.463	
Change in fish for dinner (scale)	1.135 (0.346–3.729)	0.835	1.656	
Change in butter/margarine on bread (scale)	**0.030 (0.001–0.652)**	**0.026**	1.198	
Change in noodles (scale)	0.379 (0.117–1.228)	0.106	1.845	
Parsimonious model				
Age (years)	**0.578 (0.399–0.837)**	**0.004**	1.015	0.521
Change in butter/margarine on bread (scale)	**0.097 (0.019–0.512)**	**0.006**	1.022	
Change in noodles (scale)	**0.573 (0.329–0.999)**	**0.049**	1.021	

ANR, adenoidal-nasopharyngeal ratio; CI, confidence interval; OSA, obstructive sleep apnea; REM, rapid eye movement; SpO_2_, peripheral oxygen saturation; VIF, variance inflation factor. Bold font indicates statistically significant differences (*p* < 0.05).

## 4. Discussion

The current study demonstrated several novel and interesting findings on the dietary profiles of pediatric OSA patients, their differences compared to healthy children without OSA, the effectiveness of routine educational counseling, and predictors for cured OSA among patients receiving combined treatment.

The most commonly consumed food items/groups among pediatric OSA patients were full-fat milk, vegetables, rice, fruit/berries, noodles, fish for dinner, and sweets, and they had fruit drinks with sugar, vegetables, sweets, chocolate, rice, and noodles more frequently than the healthy control did. Daily fruit/berry consumptions in Group 1 and the Control Group were lower than the Taiwanese recommendation (3.5 servings per day) (37, 45). This is not uncommon in Taiwan; although healthy Taiwanese children have a higher median daily vegetable consumption than that of normal Norwegian children (37), the value is still lower than the recommendation (4–5 servings per day) from the Daily Dietary Guidelines of Taiwan for children aged 7–12 years (45). Likewise, Taiwanese children in general drink less full-fat milk than the recommended (1.5 glasses of milk/day) (45). Some child caregivers of our subjects reported that they reduced full-fat milk consumption and replaced it with low-fat milk to lower the risk of childhood obesity. The impacts of dairy foods and their low-fat products on cardio-metabolic health are still controversial, called the “dairy fat paradox” (52). Vanderhout et al. demonstrated that higher cow milk fat intake was related to lower childhood adiposity in a meta-analysis (53). However, most previous research focused on cardiovascular diseases, metabolic syndrome, or weight status as study outcomes; the role of full-fat milk consumption in pediatric OSA is still unclear and needs further investigation.

Consistent with Spruyt’s study (47), the pediatric OSA patients with obesity in the present study ate more fast food and less healthy food, such as vegetables and fruits, than the children without OSA. Elevated ghrelin levels are positively associated with OSA, increased appetite and caloric intake in children with obesity (47). On the contrary, more frequent fruit consumption has been correlated with healthy sleep, including shorter nap duration (54) and long sleep duration in children (55). Our data combined with the literature indicated that education on healthy eating, such as more vegetable/fruit intake and less fast food, needs to be addressed more intensively among children in Taiwan, especially for pediatric OSA patients. The literature indicates that longer, better, and more regular sleep links to lower adiposity and better metabolic health (16). The educational counseling delivered in this study focused firstly on sleep hygiene, which was not only meant to directly improve the sleep and circadian rhythm of participants, but also the efforts in theory would have positive impacts on weight status and body composition. Moreover, the counseling promoted healthy eating and regular exercise, which were both well-documented effective methods to weight management (56).

Our data suggested that pediatric OSA patients receiving combined intervention might have additional treatment benefits other than solitary adenotonsillectomy. First, they had significantly increased N2 and REM sleep proportions and reduced N3 sleep proportion, which suggested a restoration of sleep architecture from disruption toward normal (57). Second, they had a significantly higher mean SpO_2_ meant, which suggested that the sleep hypoxemia status could be improved by not only surgical treatment but also approaches to lifestyle modification such as sleep hygiene, healthy eating, and regular exercise. However, as current evidence supports that weight reduction through lifestyle and dietary interventions can improve OSA severity in adults, our routine educational counseling did not show effectiveness on significant changes in dietary behavior, weight status, or OSA severity, indicating that more intensive lifestyle interventions are needed to yield more substantial clinical outcomes.

The full multivariable logistic regression model showed that younger age and decreased use of butter/margarine on bread were significant predictors of cured OSA under the control of possible confounding factors in the full model. Older age is a well-recognized risk factor for residual OSA after adenotonsillectomy in children, together with obesity and neurological/developmental/craniofacial comorbidities (8, 11, 12). Butter and margarine are used as spreads on bread and for cooking and baking. Butter is a dairy product generated from milk, containing approximately 80% milk fat and 16% water, and is rich in saturated fats, proteins, calcium, phosphorus, and some essential fat-soluble vitamins (A, D, E) (58). Among children and adolescents, butter/margarine spread is significantly related to excessive weight gain (22). Similar to patients with adulthood OSA, improper dietary habits (such as the frequent use of butter/margarine spread or as a source of fat for cooking) has been associated with body weight gain and OSA (59). Consistent with the literature, our results suggest that a reduction in butter/margarine on bread may help weight control and decrease the severity of childhood OSA.

Interestingly, the parsimonious model further included reduced noodle consumption in addition to younger age and reduced butter/margarine on bread for predicting cured OSA. Noodles are a staple food in Asian countries, and they are made from wheat flour, water, starch, and salt. One hundred grams of noodles have approximately 138 kcal, 2.07 g fat, 25.2 g carbohydrate, 4.54 g protein, 1.2 g fiber, 67.7 g water, and 5 mg sodium (60). Excessive consumption of noodles has been linked to overweight and obesity among school-aged children and adolescents (61), and a higher intake of instant noodles may increase the risk of short sleep duration and poor sleep quality in adolescents (62). To our best knowledge, this study was the first to reveal a significant association between the consumption of noodles and OSA in children. However, since the change in noodle consumption was not related to the change in AHI, we postulated that a reduction in noodle consumption might connect to cured OSA via indirect pathways such as weight reduction.

Somatic growth and weight gain are frequently observed after adenotonsillectomy in children with or without OSA (36, 63, 64). These observations may be explained by the improved retro-nasal olfactory function and consequently increased appetite after tonsil and adenoid removal (65). We conducted routine educational counseling for pediatric OSA patients in Group 1 to promote a healthy lifestyle. Some positive impacts were observed on sleep architecture, oxygenation, and undesirable eating habits. However, item/group-specific food frequencies did not change significantly after a 12-month follow-up, suggesting more intensive educational counseling was needed to yield profound change in food literacy. A multidisciplinary team, including family physicians, nurses, clinical dietitians, social workers, and administrative employees from schools, with a longer interventional duration, may be more effective (66). Besides, like multidisciplinary weight loss interventions in youth (10–19 years old) with obesity, educational counseling with more sessions (10–20 times) for a more extended period (4–12 months) may be more likely to result in improvement of clinical outcomes such as weight status and disease severity.

The greatest strength of this investigation was the inclusion of both a sample of representative and well-characterized pediatric OSA patients and a large sample of healthy children as the control. Furthermore, a long-term follow-up after adenotonsillectomy was completed for pediatric OSA patients. This design allowed a detailed description of diet behaviors across several factors, which we co-adjusted to identify independent associations with cured OSA. Nevertheless, these results need to be interpreted in consideration of some limitations. First, the completeness rate of Group 1 was lower than anticipated (76%), which might be attributed to the outbreak of COVID-19 in Taiwan during the study period. Second, a high degree of heterogeneity in the study groups might lead to increased type II errors due to a lack of statistical power (range: 36–99%), especially in comparing the intervention success group and the residual disorder group in Group 1. Third, the number of routine educational sessions conducted in our study was limited. As previously acknowledged, a larger number of sessions with an extended duration may be more effective in inducing substantial dietary modifications. Forth, although the SFFQ has been reported comparable to the four-day recoded food diary (regarding consumption frequencies and mean intake of food) and the 24-h dietary recall (regarding nutrients intake) (67), this western-country developed questionnaire may not be able to fully reflect the food literacy of Asian children. The lack of change in food frequencies could be a result of the ineffectiveness of the educational counseling, but it could also be due to methodological limitations. Furthermore, tastes/interests in young children vary frequently; other food frequency questionnaires with shorter intervals may reflect more recent diet habits without recall bias. Lastly, the role of sleep hygiene in food preferences, weight status, and OSA severity are not well-established and needs to be further delineated. Nevertheless, our preliminary findings warrant future investigations; a more intricate case–controlled study or a randomized controlled trial with a larger sample will be of interest.

In conclusion, the present study preliminarily characterized a relatively unhealthy dietary profile in pediatric OSA patients compared with the healthy control or the domestic daily dietary recommendations. The results showed that educational counseling in routine workups led to subjective and objective improvements in sleep outcomes after adenotonsillectomy; however, food consumption frequency was not changed substantially. Consumption frequently of certain food items/groups, including butter/margarine on bread and noodles, may be involved in the resolution of OSA after intervention. Given the potential beneficial effects of nutritional interventions on weight control and OSA amelioration, approaches including these item/group-specific foods could be a strategy for the comprehensive management of childhood OSA.

## Data availability statement

The original contributions presented in the study are included in the article/supplementary material, further inquiries can be directed to the corresponding author.

## Ethics statement

The study involving human participants was reviewed and approved by the Institutional Review Board of the Chang Gung Medical Foundation, Taoyuan, Taiwan (202000873B0 and 202201649B0). Written informed consent from the patients/participants legal guardian/next of kin was not required to participate in this study in accordance with the national legislation and the institutional requirements.

## Author contributions

H-HC, R-HL, J-FH, and L-AL conceived and planned the study. H-HC, J-FH, L-PC, H-YL, Y-SH, and L-AL enrolled the patients. H-HC, R-HL, J-FH, Y-SH, AY, G-SL, TK, CY, and L-AL designed the study, analyzed the data, made the statistics, and interpreted the results. H-HC, R-HL, J-FH, L-PC, and L-AL participated in manuscript drafting. H-YL, T-JF, AY, G-SL, TK, and CY supervised the study. All authors read and approved the final manuscript.

## Funding

This study was supported by the National Science and Technology Council, Taiwan (grant number 109-2314-B-182-083-MY3) and the Chang Gung Medical Foundation, Taiwan (grant numbers CMRPG3F1091-3 and CMRPG3L0811-2).

## Conflict of interest

The authors declare that the research was conducted in the absence of any commercial or financial relationships that could be construed as a potential conflict of interest.

## Publisher’s note

All claims expressed in this article are solely those of the authors and do not necessarily represent those of their affiliated organizations, or those of the publisher, the editors and the reviewers. Any product that may be evaluated in this article, or claim that may be made by its manufacturer, is not guaranteed or endorsed by the publisher.
